# Predictors of time to conversion of new‐onset atrial fibrillation to sinus rhythm with amiodarone therapy

**DOI:** 10.1002/joa3.12372

**Published:** 2020-06-01

**Authors:** Ihsan Dursun, Sinan Sahin, Ali Bayraktar, Omer Faruk Cirakoglu, Selim Kul, Levent Korkmaz

**Affiliations:** ^1^ Department of Cardiology Ahi Evren Thoracic and Cardiovascular Surgery Training and Research Hospital Saglik Bilimleri University Trabzon Turkey

**Keywords:** amiodarone, atrial fibrillation, cardioversion, epicardium

## Abstract

**Background:**

New‐onset atrial fibrillation (AF) is a frequent cause of presentation to the emergency department (ED). Epicardial fat thickness (EFT) is associated with the presence and recurrence of AF. However, no study has investigated the predictors of the time to conversion of AF to sinus rhythm with amiodarone therapy. The aim of this study was to investigate predictors of time to conversion of AF to sinus rhythm in patients with new‐onset AF.

**Methods:**

A total of 122 patients admitted to the ED with symptoms of hemodynamically stable new‐onset AF (lasting <48 hours) were registered consecutively. These patients received intravenous amiodarone. EFT was measured using 2D echocardiography in parasternal long‐axis views.

**Results:**

A significant positive correlation was determined between EFT and conversion time (rho = 0.267, *P* = .017) in all patients. The median time for conversion from the start of amiodarone infusion was 410 min (150‐830 minutes). Based on the median conversion time, patients were classified as early conversion (time < 410 minutes; n = 41) and late conversion (time > 410 minutes; n = 40). Multivariate logistic regression analysis demonstrated that EFT (*P* = .033, odds ratio [OR]: 1.68, 95% confidence interval [CI]: 1.6‐2.7), higher troponin I level > 0.04 (*P* = .034, OR: 5.3, 95% CI: 1.1‐24.8), and lower age (*P* = .003, OR: 0.8, 95% CI: 0.8‐0.9) were significantly associated with longer conversion time.

**Conclusions:**

We determined that EFT and high troponin level affected the time to conversion to sinus rhythm in patients with new‐onset AF.

## Graphical abstract

Epicardial fat thickness (EFT) is associated with the presence and recurrence of atrial fibrillation (AF). However, no study has investigatedthe predictors of the time to conversion of AF to sinus rhythm with amiodarone therapy. We determined that EFT and high troponin level affected the time to conversion to sinus rhythm in patients with new‐onset AF

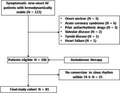

## 1 INTRODUCTION

Atrial fibrillation (AF) is one of the leading causes of admission to emergency department (ED) worldwide and is the most frequently treated arrhythmia in ED.^1^, ^2^ AF is associated with an increased risk of thromboembolism, congestive heart failure, and all‐cause mortality.^3^ The treatment goals during the acute setting of AF are control of symptoms, hemodynamic stabilization, reduction in atrial remodeling, reduction in thromboembolic complications, and reduction in hospital stay.^4^, ^5^, ^6^, ^7^ Amiodarone is a class III antiarrhythmic agent that has been used extensively for the pharmacological management of AF in the ED.^5^, ^8^, ^9^ Amiodarone is the most commonly used rhythm control agents in our country because the intravenous forms of drugs recommended in the guidelines^10^ are not available in our country.

Epicardial fat (EpF) is the visceral fat deposit located between the myocardium and visceral layer of the serous pericardium that has metabolic and inflammatory properties. Epicardial adipose tissue is an essential source of various pro‐inflammatory and pro‐atherogenic factors, such as leptin, IL‐6, adipocytokines, and TNF‐alpha.^11^ The pathogenesis of AF is complex and there are several risk factors for developing AF.^12^ EpF has been identified as a novel risk factor for AF over the last decade and may lead to AF through the structural and electrical remodeling of the atria through the infiltration of adipose tissue by acting as a source for paracrine modulators of myocardial inflammation and oxidative stress.^13^, ^14^ In addition, recent studies have reported an association between EFT and AF recurrence and success of electrical cardioversion.^15^, ^16^

Because the conversion time with amiodarone is longer than with other antiarrhythmic drugs,^7^, ^9^, ^17^ the determination of early conversion predictors is crucial in terms of length of hospital stay. Most previous studies regarding the pharmacological cardioversion of AF have focused on conversion rates of drugs and time to conversion.^7^, ^18^, ^19^ However, none of these studies have addressed factors affecting the conversion time. Therefore, in this study, we aimed to investigate the factors affecting the conversion time in pharmacological cardioversion of new‐onset AF with amiodarone therapy.

## 2 METHODS

### 2.1 Study design and population

**FIGURE 1:**
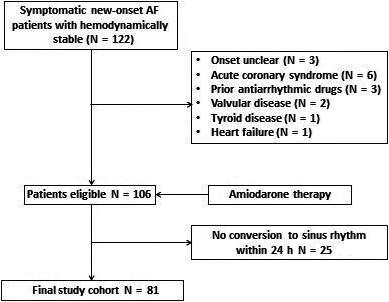
A flowcharts of the study

The study included 122 patients diagnosed with new‐onset AF (lasting <48 hours) admitted to the ED between August 2017 and June 2018. In particular, symptomatic patients with an onset of AF less than 48 hours, above 18 years of age, and a well‐defined onset of AF were included in the study (Figure 1). All patients were hemodynamically stable.

The exclusion criteria were as follows: significant heart failure, hemodynamic instability, acute coronary syndromes at the time of admission and in the preceding month, cardiac surgery in the previous three months, prior antiarrhythmic therapy with class I or III antiarrhythmic drugs, intracardiac thrombus, significant valvular heart disease, patients who did not convert to sinus rhythm within 24 hours of treatment initiation, history of catheter ablation, thyroid illness, pregnancy, patients whose AF onset time remains unclear, pulmonary fibrosis, renal or liver insufficiency, stroke, transient ischemic attack or thromboembolism within the previous three months, Wolff‐Parkinson‐White syndrome, and known sick sinus syndrome. Even if cardiac troponin found elevated, acute coronary syndrome was ruled out when the absence of an acute ST‐segment elevation or depression and ongoing chest pain. If the presence suspicion of ischemia we performed coronary angiography to rule out acute coronary syndrome. The study protocol was approved by the Institutional Ethical Committee, and written informed consent was obtained from all patients in advance.

The study excluded 25 patients who had not converted to sinus rhythm 24 hours after the initiation of amiodarone therapy. Finally, after excluding 41 patients based on the exclusion criteria, 81 consecutive patients (37 men, median age 62 years [interquartile range [IQR]: 53‐69 years]) with new‐onset AF lasting <48 hours were enrolled in the study.

The diagnosis of AF was confirmed by the absence of consistent P waves and the presence of irregular RR intervals on the electrocardiogram. Patients underwent continuous monitoring for heart rhythm and blood pressure during the observation period. As soon as sinus rhythm was restored, a 12‐lead electrocardiogram was obtained, and sinus rhythm was confirmed. Generally, patients who converted to sinus rhythm were discharged 2 hours later.

The baseline clinical characteristics, including age, gender, body mass index, history of congestive heart failure, hypertension, diabetes mellitus, thromboembolic events, vascular disease, and the presence of obstructive sleep apnea were recorded, and CHA_2_DS_2_‐VASc scores were calculated.

### 2.2 Amiodarone therapy

The dose of intravenous amiodarone for the conversion of AF remains unclear, and different dose protocols have been applied in previous studies. In this study, we followed the ACC/ AHA/ HRS 2014 guidelines regarding the treatment of AF and our amiodarone dosage protocol was applied for this guideline.^10^ Amiodarone was administered as an intravenous bolus of 150 mg in 10 minutes, followed by a continuous infusion of 1 mg/min for 6 hours, then 0.5 mg/min for 18 hours.^10^ The amiodarone infusion was stopped as soon as sinus rhythm was restored. The time to conversion to sinus rhythm from the start of amiodarone infusion was recorded. If AF was still present after 24 hours of amiodarone infusion, all eligible patients underwent electrical cardioversion. Patients who underwent electrical cardioversion were subsequently excluded from the study. Patients received unfractionated heparin at a dosage of 100 IU/kg. Rate control drugs (beta‐blockers, nondihydropyridine calcium channel blockers) were administered at the discretion of the physician in case of ongoing tachycardia after administration of the bolus dose of amiodarone.

### 2.3 Echocardiographic examinations

Transthoracic echocardiographic (TTE) examination was performed using the Vivid 5 system (GE Vingmed Ultrasound AS) unit with a 2.5 MHz probe. The conventional B‐mode parameters and ejection fraction were measured according to the American Society of Echocardiography guidelines.^20^ Echocardiographic examination was performed at the admission to measure EFT and after conversion to evaluate significant valvular pathologies and ejection fraction measurements more accurately. All echocardiographic measurements were performed by the same operator. In the event of suspicious intracardiac thrombus, transesophageal echocardiography was performed. EFT—identified as an echo‐free space between the myocardium and pericardium—was measured on the right ventricular free wall in parasternal long‐axis views at the end‐systole, using the mean of three consecutive beats.^11^

### 2.4 Blood tests

Venous blood samples were obtained on admission, and the total leukocyte count and cardiac enzymes levels were recorded. We measured the value of cardiac troponin I (cTnI) to exclude acute coronary syndrome in patients with new‐onset AF and evaluate the independent correlation of high cTnI levels to conversion time. CTnI level was obtained both upon admission and four hours later. The upper reference limit of the cTnI assay of our laboratory was ≥0.04 ng/mL. A cTnI elevation above 99th percentile of upper reference limit was defined as high cTnI level.

### 2.5 Statistical analysis

Continuous variables were expressed as mean ± SD and median (IQR) according to their distribution. Categorical variables were expressed in numbers and percentages. Baseline clinical characteristics and other categorical variables of patients were compared using the Chi‐squared test. Parametric variables were analyzed using independent sample *t*‐test, and nonparametric variables were analyzed using the Mann‐Whitney U test. Multiple logistic regression was performed to assess the independent predictors of early conversion time. Multiple logistic regression was performed using the median time to conversion of AF as the dependent variable, and clinical, echocardiographic, and angiographic variables as the covariates. The included covariates were age (continuous variable), epicardial fat thickness (EFT; continuous variable), gender, history of diabetes, history of hypertension, BMI > 30 kg/m^2^, high cTnI level, rate control drugs, left atrial diameter (continuous variable), and more than one AF attack. Spearman's correlation analysis was performed to investigate the association between EFT and time to conversion of AF *P* < .05 (two‐tailed) were considered significant. Data were analyzed using the SPSS 18.0 statistical software (SPSS Inc).

## 3 RESULTS

Baseline clinical and demographic characteristics of patients are as shown in Table 1. The median age was 62 years (53‐69 years). Patients had high hypertension rates (61%) and BMI levels (29.7 kg/m^2^). In 40 (49%) patients, the AF episode was the first episode, whereas the remaining patients had a history of two or more episodes. Forty‐nine patients (61%) had a CHADSVASc score of 2 or more. The mean heart rate on admission was 138 ± 21 bpm, and the median value of systolic blood pressure was 130 mm Hg (110‐140 mm Hg). A total of 42 patients received rate control drugs. Notably, metoprolol (n = 16) and diltiazem (n = 26) were mostly used. No serious side effects were observed with amiodarone, and none of the patients had thromboembolic complications related to pharmacological conversion. A 50% reduction in admission heart rate was observed after the restoration of sinus rhythm.

**TABLE 1:** Clinical and laboratory parameters in patients stratified by time to conversion

Variable	All patients	Time to conversion		*P* value
		<410 min (n = 41)	>410 min (n = 40)	
Age, y	62 (53‐69)	64 (57‐69)	62 (46‐69)	.086
Male sex, (n, %)	37 (46)	20 (49)	17 (43)	.570
BMI (kg/m^2^)	29 (27‐36)	29 (26‐36)	31 (28‐34)	.473
Hypertension (n, %)	49 (61)	23 (56)	26 (65)	.413
Diabetes (n, %)	17 (21)	10 (24)	7 (18)	.446
Stroke/TIA (n, %)	6 (7)	2 (5)	4 (10)	.432^a^
OSAS (n, %)	4 (5)	3 (7)	1 (3)	.616^a^
Episode > 1 (n, %)	40 (51)	22 (54)	18 (45)	.436
CHADS‐VASc score > 1 (n, %)	49 (61)	26 (63)	23 (59)	.684
Duration of AF (min)	230 (60‐705)	220 (60‐585)	235 (48‐720)	.633
Systolic blood pressure (mm Hg)	130 (110‐140)	120 (110‐140)	130 (117‐140)	.496
Ventricular rate, admission (bpm)	138 ± 21	138 ± 12	137 ± 22	.802
Ventricular rate, sinüs (bpm)	68 ± 10	68 ± 9	68 ± 11	.828
Rate control drug (n, %)	41 (51)	20 (49)	21 (54)	.650
EPF thickness (mm)	4.8 ± 1.3	4.5 ± 1.2	5.1 ± 1.4	.079
WBC (/µL*1000)	8.3 ± 2.3	8.2 ± 2.4	8.4 ± 2.1	.744
High Troponin‐I level (n, %)^b^	12 (15)	4 (10)	8 (22)	.217^a^
LV Ejection fraction (%)	66 (64‐69)	66 (61‐69)	67 (64‐69)	.404
Left atrial size (mm)	35 (31‐39)	35 (31‐39)	35 (32‐38)	.974

Note

Values are mean ± SD, median (interquartile range).

Abbreviations: AF, atrial fibrillation; BMI, body mass index; LV, left ventricular; OSAS, obstructive sleep apnea syndrome; TIA, transient ischemic attack; WBC, white blood count.

^a^ Fischer exact test.

^b^ Troponin‐I level > 0.04 ng/mL.

A significant positive correlation was observed between EFT and conversion time (rho = 0.267, *P* = .017) in all patients. However, no correlation was observed between EFT and left atrial size (*P* = .648) and BMI (*P* = .959).

**FIGURE 2:**
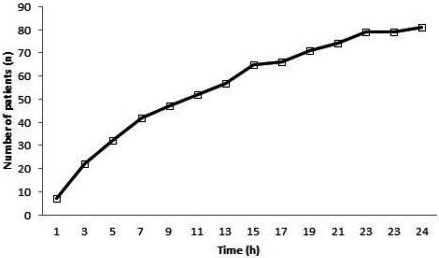
The number of patients who converted to sinus rhythm from the onset of amiodarone therapy

The median time of symptom duration before admission was 230 minutes (60‐705 minutes). The median time to conversion to sinus rhythm from the start of amiodarone infusion was 410 minutes (150‐830 minutes; Figure 2). Based on the median conversion time, patients were grouped as early conversion (time < 410 minutes; n = 41) and late conversion (time > 410 minutes; n = 40). Baseline clinical, echocardiographic, and laboratory characteristics of patients stratified based on the conversion time are as shown in Table 1. No significant intergroup differences were observed regarding clinical, echocardiographic, and laboratory variables. Mean EFT was lower in patients with early conversion time, albeit not statistically significant (4.5 ± 1.2 mm vs 5.1 ± 1.4 mm, respectively; *P* = .0679). Predictors of early pharmacological conversion were assessed using multivariate logistic regression analysis. Multivariate logistic regression analysis showed that a high EFT (*P* = .033, odds ratio [OR]: 1.6, 95% confidence interval (CI): 1.6‐2.7), higher troponin *I* level > 0.04 (*P* = .034, OR: 5.3, 95% CI: 1.1‐24.8), and lower age (*P* = .003, OR: 0.8, 95% CI: 0.8‐0.9) were significantly associated with longer conversion time (Table 2).

**TABLE 2:** Predictors for the time to conversion of AF‐treated amiodarone

Variables	Odds ratios, ( 95% CI)	*P* value
Male sex	1.7 (0.3‐8.1)	.497
Hypertension	0.3 (0‐1.6)	.179
Age	0.8 (0.8‐0.9)	.003
Diabetes	1.2 (0.2‐5.1)	.772
LA size	1 (0.9‐1.1)	.748
EPF thickness	1.6 (1.04‐2.73)	.033
Obesity^a^	0.9 (0.2‐3.5)	.967
Episode > 1	1.7 (0.5‐5.9)	.354
High troponin level^b^	5.3 (1.1‐24.8)	.034
Rate control drug	1.2 (0.4‐3.6)	.736

^a^ BMI > 30 kg/m^2^.

^b^ Troponin‐I level > 0.04 ng/mL.

## 4 DISCUSSION

This study was conducted to detect the predictors of time to conversion in patients with new‐onset, uncomplicated AF treated with pharmacological conversion using amiodarone in the ED. The present study is the first to evaluate the predictors of time to conversion to sinus rhythm with intravenous amiodarone in patients with acute‐onset AF. Our results show that EFT, age, and high cTnI levels were independently associated with time to conversion.

The association between epicardial adipose tissue and AF has already been reported, with its paracrine and vasocrine functions reportedly playing a role in this setting.^13^, ^14^ According to previous studies, epicardial adipose tissue seems to be intimately bound to AF physiopathology, even though the precise mechanisms are yet to be elucidated.^12^, ^13^ In addition, the relationship between EFT and AF incidence and that between EFT and AF recurrence has been reported in recent years, both after ablation and electrical cardioversion.^14^, ^15^, ^16^, ^21^, ^22^ Although no significant difference was observed regarding the median EFT between early and late conversion groups, multivariate regression analysis revealed that EFT was an independent predictor of conversion time. Dereli et al recently reported the relationship between EFT and cardioversion success in patients with AF. They showed that EFT was an independent predictor of electrical cardioversion success and a crucial predictor of six‐month recurrence in patients with persistent AF.^15^ Our study is the first clinical study to evaluate the effects of EFT on time to conversion of new‐onset AF to sinus rhythm. The results of this study suggest that EFT can significantly affect the time to conversion to sinus rhythm during pharmacological cardioversion with amiodarone. EFT is simple to detect and can be identified using TTE. Moreover, TTE can be performed easily in patients with new‐onset AF and can contribute to the decision‐making process regarding the continuation of amiodarone therapy.

Obesity has been shown as an independent risk factor for AF.^12^, ^23^ However, the association between EpF and AF has been observed to be independent of obesity.^21^, ^24^ Despite a higher obesity rate in patients with AF, in our study, no correlation was observed between BMI and EFT. Moreover, multivariate regression analysis revealed that the association between time to conversion and EpF was independent of BMI.

Even though amiodarone is not recommended as the first choice for pharmacological cardioversion in acute‐onset AF as per the published guidelines of AF treatment,^10^ it is still the first choice in ED worldwide.^5^, ^9^, ^25^ Notably, amiodarone is the preferred drug for pharmacological cardioversion of AF in the ED in the United States (22%), the United Kingdom (48%), and Australia (63%).^3^ In our country, amiodarone is the only intravenous drug used in ED because other intravenosus drugs, such as flecainide, ibutilide, propafenone, and dofetilide are not available, which are recommended for the pharmacological cardioversion. The mean conversion time with amiodarone was reported to be ranging from 224 to 414 minutes,^4^, ^18^, ^19^, ^26^ which was consistent with the median time to conversion of 410 minutes in our study.

In recent years, most of the studies on AF have been related to catheter ablation strategies and new oral anticoagulant therapies. To the best of our knowledge, no previous study has revealed any information regarding predictors of early conversion time after amiodarone therapy in new‐onset AF. In a study by Zahir and Lheureux^27^ that analyzed the factors associated with early successful cardioversion in the ED, the factors associated with early conversion to sinus rhythm were age <65 years and symptom duration of <48 hours. However, patients in this study were treated not only with amiodarone but also other antiarrhythmic drugs and electrical cardioversion. In addition, this study identified no specific time frame for early conversion.^27^ Galve et al^18^ reported that the absence of congestive heart failure, smaller left atrial size, and absence of previous history of supraventricular arrhythmias were independent predictors of conversion time to sinus rhythm during amiodarone therapy.

Cardioversion with amiodarone therapy results in an extended hospital stay compared with other treatment options.^4^, ^6^, ^7^, ^19^ The unpredictable duration of sinus rhythm restoration may affect the length of stay in the ED and may result in overcrowding. Overcrowding in ED has been seen as a critical issue affecting the treatment of diseases.^6^, ^28^ Therefore, the early identification of clinical factors related to conversion time to sinus rhythm of new‐onset AF in patients who received amiodarone could help physicians in curtailing the overcrowding in the ED.

In our study, we also determined that high troponin levels are related to the conversion time. Elevated concentrations of cTnI have been observed in patients with AF in the absence of acute coronary syndrome.^19^, ^29^ In this study, patients with an acute coronary syndrome were excluded. In addition, an acute coronary syndrome was ruled out through coronary angiography when ischemia was suspected in patients with high cTnI. Although the potential mechanism of troponin elevation in the setting of AF is not well‐defined, it could be probably due to tachycardia‐induced ischemic stress.^29^, ^30^, ^31^ Conti et al^32^ demonstrated that in patients presenting acute AF, even minor cTnI elevations could predict adverse events and add prognostic information over the clinical parameters. The ARISTOTLE (Apixaban for the Prevention of Stroke in Subjects with Atrial Fibrillation) study^29^ results showed that the troponin levels were independently associated with an increased risk of stroke, cardiac death, and major bleeding and can improve risk stratification. Therefore, high cTnI levels can help predict the conversion time in patients in whom high troponin levels are not considered to be associated with an acute coronary syndrome.

The prevalence of AF increases with age.^12^ In our study, we found that advanced age was related to early conversion time. This finding could be attributed to the higher incidence of symptoms associated with AF in young patients, as well as related to increased anxiety. It has been reported that palpitations are more common in patients with AF below 65 years of age.^33^

### 4.1 Limitation

Nonetheless, our study had limitations. We only evaluated factors affecting the duration of cardioversion and did not evaluate the safety and efficacy of intravenous amiodarone therapy. Therefore, amiodarone therapy cannot be recommended as the best approach for pharmacological cardioversion of acute AF in the ED. Vernakalant is a recommended drug for cardioversion in recent guideline.^34^ But vernakalant was not available in our country. Our study had no data regarding inflammatory markers, such as C‐reactive protein and cytokines. Notably, measurement of inflammatory markers, in addition to EFT, could have strengthened our study. In our study, systemic adiposity components, such as abdominal fat and waist circumference, were not measured. Systemic adiposity was only evaluated based on body mass index, and it was added to the regression analysis as a covariate. Another limitation of the study was that EFT measurement was only performed using TTE, and its accuracy was not supported by other imaging techniques, such as computerized tomography, magnetic resonance imaging. Lastly, our study had a small sample size. The multivariate analysis was performed using 10 variables. However, only 41 patients had an early conversion to sinus rhythm. But, there should not be less than 10 events for each variable. Therefore, this warrants larger prospective studies exploring the association between EFT and conversion time.

## 5 CONCLUSION

Our study suggests that EFT and high cTnI levels were independently associated with time to conversion to sinus rhythm during pharmacological cardioversion with amiodarone. Therefore, EFT detected using echocardiography may help in the decision‐making process regarding initiation or continuation of amiodarone therapy for cardioversion in patients with new‐onset AF because this decision affects the length of stay in the ED. In addition, longer conversion times extend the in‐hospital stay length, thereby affecting medical care and costs.

## CONFLICT OF INTEREST

The authors declared no potential conflicts of interest with respect to the research, authorship, and/or publication of this article.

## AUTHORS' CONTRIBUTIONS

ID, SS, and A.B involved in concept/design and data collection. ID and S.S also performed data analysis/interpretation. All authors discussed the results and contributed to the final manuscript.
